# Female Gender Is Associated with Higher Susceptibility of Weight Induced Arterial Stiffening and Rise in Blood Pressure

**DOI:** 10.3390/jcm10163479

**Published:** 2021-08-06

**Authors:** Junli Zuo, Huijuan Chao, Biwen Tang, Alberto P. Avolio, Markus P. Schlaich, Janis Marc Nolde, Audrey Adji, Revathy Carnagarin

**Affiliations:** 1 Department of Geriatrics and Geriatrics Centre, Ruijin Hospital/Jiaotong University School of Medicine, Shanghai 200240, China; zjl12616@rjh.com.cn (J.Z.); chj12752@rjh.com.cn (H.C.); tbw12673@rjh.com.cn (B.T.); 2 Department of Biomedical Sciences, Faculty of Medicine, Health and Human Sciences, Macquarie University, Sydney, NSW 2109, Australia; alberto.avolio@mq.edu.au; 3 Dobney Hypertension Centre, School of Medicine—Royal Perth Hospital Research Foundation, University of Western Australia, Perth, WA 6000, Australia; markus.schlaich@uwa.edu.au (M.P.S.); janis.nolde@uwa.edu.au (J.M.N.); 4 Departments of Cardiology and Nephrology, Royal Perth Hospital, Perth, WA 6000, Australia; 5 Neurovascular Hypertension & Kidney Disease Laboratory, Baker Heart and Diabetes Institute, Melbourne, VIC 3004, Australia; 6 St Vincent’s Hospital and Clinical School UNSW, Sydney, NSW 2000, Australia; 7 Victor Chang Cardiac Research Institute, Sydney, NSW 2010, Australia

**Keywords:** obesity, hypertension, body mass index, pulse wave velocity, arterial stiffness

## Abstract

Arterial stiffness is an important predictor of cardiovascular events, independent of traditional risk factors. Stiffening of arteries, though an adaptive process to hemodynamic load, results in substantial increase in the pulsatile hemodynamic forces that detrimentally affects the microcirculation perfusing the vital organs such as the brain, heart and kidneys. Studies have proposed that arterial stiffness precedes and may contribute to the development of hypertension in individuals with obesity. Our study sought to determine the gender-based effects on arterial stiffening in obesity which may predispose to the development of hypertension. We found female sex is associated with higher susceptibility of weight-related arterial stiffening and rise in blood pressure in obesity. Women had significantly higher carotid-femoral pulse wave velocity (CF-PWV) with higher body mass index (BMI) status (normal: 7.9 ± 2 m/s; overweight: 9.1 ± 2 m/s; obese: 9 ± 2 m/s, *p* < 0.001), whereas it was similar in males across all BMI categories. The linear association between arterial stiffness and BMI following adjustment for age and brachial systolic and diastolic blood pressure (BP), remained significant in females (β = 0.06; 95% CI 0.01 to 0.1; *p* < 0.05) but not in males (β = 0.04; 95% CI −0.01 to 0.1; *p* > 0.05). The mean CF-PWV values increased by 0.1 m/s for every 1 kg/m^2^ increase in BMI in the female subjects in the age adjusted linear model, while such effect was not seen in the male subjects. In line with arterial stiffening, the overweight and obese females demonstrated significantly higher systolic brachial BP. (BP difference: ΔBP 9−11 mmHg, *p* < 0.01) and central systolic pressure (ΔBP 8−10 mmHg, *p* < 0.05) compared to their lean counterparts, unlike the male subjects. Our results suggest that female gender is associated with higher susceptibility of weight-related arterial stiffening and rise in blood pressure.

## 1. Introduction

Obesity is a pandemic on the fast track, associated with adverse cardiovascular (CV) outcomes in both genders. Hypertension is an increasingly prevalent risk factor that often coexists with obesity in both men and women, mostly attributable to the increasing obesity prevalence [1,2]. Recent trends reveal that ~70% of arterial hypertension is associated with obesity [1]. Elevation in arterial stiffening is a marker of vascular target organ damage (TOD) and has emerged as an independent predictor of future cardiovascular events [3,4]. Stiffening of the arterial wall and earlier return of the reflected pressure pulse wave are key determinants for elevation in systolic blood pressure (BP) at the central level, resulting in the detrimental CV outcomes independent of peripheral BP [3]. 

Arterial stiffness has been identified to precede and contribute to the development of hypertension in the general population [5], and arterial stiffness mediated hemodynamic changes have been implicated in the development and progression of hypertension [6,7]. The pathophysiological mechanisms that link obesity and arterial stiffening remain incompletely understood. However, obesity is associated with vascular remodelling and stiffness that has been shown to predict CV mortality and morbidity in obesity [8]. Mechanisms such as insulin resistance, hyperleptinemia, enhanced inflammatory mediators such as uric acid levels and free fatty acids in the circulation, as well as mechanical shear stress on arterial walls owing to obesity mediated volume overload are some of the proposed mechanisms of obesity-mediated vascular TOD [9,10,11,12]. Furthermore, obesity is associated with heightened sympathetic activation [13], while reversal of arterial stiffening has been demonstrated in parallel to the reduction in heart rate, following weight loss [14,15,16]. Moreover, reduced elasticity has been observed in both central and peripheral arteries in obesity [17].

The risk of obesity–related hypertension is sex specific [18,19]. Population rates of obesity are higher in women than men with its prevalence and severity being much higher in women, across all nations despite the socioeconomic status [20]. In addition, emerging data suggest a disproportionate impact of obesity on arterial hypertension and CV health in women compared to men [21] with stronger association between hypertension and obesity in women [22,23]. Moreover, women have higher lifetime risk of hypertension, with obesity cited as the most significant risk factor [24]. Furthermore, adequate blood pressure (BP) control is less likely to be achieved in obese women than men [25,26]. Hence, our study sought to determine the association of sex with weight related vascular TOD in a normal healthy cohort. 

## 2. Methods:

### 2.1. Patient Cohort

We performed a cross-sectional analysis of prospectively collected data from 836 otherwise healthy individuals attending a health assessment clinic for CV disease screening at Ruijin Hospital North, Shanghai, China, between December 2017 and September 2019. Subjects with any history of occlusive arterial disease history such as myocardial infarction or acute coronary syndrome, transient ischemic attack or stroke were excluded from the study as were patients age <18 years of age. This study was performed in accordance with the Declaration of Helsinki and the principles of Good Clinical Practice guidelines. All patients provided written, informed consent to participate in this systematic prospective data collection, which was approved by the Ethics Committee of Shangai Xuhui Central Hospital, Shanghai (approval no: 2011-30).

### 2.2. Clinical Workup

All patients had their medical history taken, underwent physical examination and collection of anthropometric data. Body height and weight were measured without shoes, waist and hip circumferences were measured with a tape while standing; waist at mid-point between the lowest rib margin and iliac crest, hip at the widest part of pelvis. Anthropometric measures such as body mass index (BMI), ratio of waist-to-hip circumference (WHR) and ratio of waist circumference to body height (WHtR) were calculated using these anthropometrics data. The subjects had their BP measured at the right arm in supine position using Omron device (BP-203RPEIII VP-1000 Kyoto, Japan), following 10 minutes rest in a quiet room with a controlled temperature of 22 °C. Carotid femoral pulse wave velocity (CF-PWV) was performed using SphygmoCor CVMS system (AtCor Medical Pty Ltd, Sydney, Australia) as per the manufacturer’s protocol in a supine position. Radial artery pressure waveforms were recorded using the high-fidelity tonometer for at least 10 s, until a stable radial tonometric pressure trace was obtained. Using the SphygmoCor CVMS system, these radial waves were calibrated to brachial cuff systolic and diastolic pressure, derived to central aortic pressure waveforms using a validated transfer function that averaged over 10 cardiac cycles to account for respiratory variation and the central pressure indices such as the central systolic and diastolic pressure, end systolic pressure, systolic ejection duration, and central augmentation index were calculated as per the manufacturer’s protocol.

***Biochemistry:*** All biochemical analysis was performed in hospital/clinic laboratory using standard methods and reagents. Full blood count, fasting glucose, lipid profile: total cholesterol (TC), triglycerides (TGL), high-density lipoproteins (HDL); renal: blood urea nitrogen, creatinine; and liver parameters: alanine transaminase (ALT), aspartate transaminase (AST) and gamma-glutamyl transferase (GGT) were determined from the same fasting venous blood samples. Serum uric acid was used as a marker of vascular inflammation [27]. LDL- cholesterol was calculated using Friedewald formula.

### 2.3. Statistical Analysis

In the cross-sectional study, all continuous variables were expressed as mean ± SD. We used one-way ANOVA and post hoc analysis, where significant to present the clinical features, anthropometric measures, arterial and biochemical parameters across the BMI groups. Regression models, adjusted for age, brachial systolic and diastolic BP and blood glucose were used to assess the gender based effect on the association of BMI with CF-PWV. Linear predictive margin analysis was used to determine the influence of gender on CF-PWV in the age-adjusted model. Pearson’s correlation analysis was used to analyse the gender-based associations of the central anthropometric measures with arterial pressure indices. A *p* value of < 0.05 was considered to be of statistical significance. Statistical analyses were performed using Stata/SE 15.1 for Windows (STATACorp LLC, College Station, TX, USA).

## 3. Results

### 3.1. Baseline Characteristics

The clinical characteristics of the study population (*n* = 834) are shown in Table 1. The mean ± SD age of participants was 54 ± 15 years and there was a greater proportion of males (*n* = 525, 63%) than females (*n* = 309, 37%). The cohort had an average height of 168 ± 8.7 cm, weight 72 ± 14kg and BMI of 29.2 ± 11 kg/m^2^. The brachial BP averaged 134/77 mmHg, central BP averaged 122/78 mmHg and the CF-PWV averaged 8.5 m/s. Subjects were stratified into 3 groups on the basis of their BMI according to WHO recommendations in the Chinese population-calculated as the weight in kilograms divided by height in meters squared (kg/m^2^) (BMI < 24 kg/m^2^: *lean*; 24−28 kg/m^2^: *overweight*, ≥28 kg/m^2^: *obese*). The study cohort included smokers (*n* = 46, 6%), type 2 diabetics (*n* = 2, 0.2%), BP controlled on antihypertensives (*n* = 158, 19%), participants who consumed alcohol (*n* = 466, 56%) and those on aspirin (*n* = 4, 0.5%) and statins (*n* = 5, 0.6%). Three hundred and six subjects (37%) had normal weight with 142 males (46%) and 164 females (53%), 345 were overweight (41%) with 244 males (71%) and 100 females (29%), 183 were obese (22%) with 138 males (75%) and 45 females (25%). Subject characteristics grouped by their BMI, age and sex are shown in Table 1 and Table 2.

### 3.2. Association of the Female Gender with Obesity Mediated Vascular TOD

Aortic stiffness was assessed by CF-PWV (m/s) across the BMI groups in both genders. PWV was high in overweight and obese females compared to their lean counterparts (normal BMI: 7.9 ± 2 m/s; overweight: 9.1 ± 2 m/s; obese: 9 ± 2 mmHg, *p* < 0.001) whereas it was similar in males across all BMI categories: normal BMI: 8.7 ± 2.4 m/s; overweight: 8.5 ± 2.1 m/s; obese: 8.5 ± 2 m/s. Linear regression analysis showed that the weight is significantly associated with CF PWV in females (*β* = 0.02; 95% CI 0.004 to 0.04; *p* < 0.05) but not in males (*β* = 0.01; 95% CI −0.004 to 0.02; *p* > 0.05) in the age and BP adjusted model. The linear association between arterial stiffness and BMI following adjustment for age and brachial systolic and diastolic BP, remained significant in females (β = 0.06; 95% CI 0.01 to 0.1; *p* < 0.05) but not in males (β = 0.04; 95% CI −0.01 to 0.1; *p* > 0.05). Similarly, the association between arterial stiffness and BMI following adjustment for age and blood glucose levels remained significant in females (β = 0.06; 95% CI 0.01 to 0.1; *p* < 0.05) but not in males (β = 0.05; 95% CI −0.002 to 0.1; *p* > 0.05). In the same model, serum uric acid (UA) levels, measured as an indicator of the pro-inflammatory milieu, was significantly associated with CF-PWV in female subjects (CF PWV: β = 0.003; 95% CI 0.00 to 0.005; *p* < 0.05) whereas this was not the case in male subjects (CF PWV: β = 0.002; 95% CI −0.00 to 0.03; *p* > 0.05).

**Figure 1:**
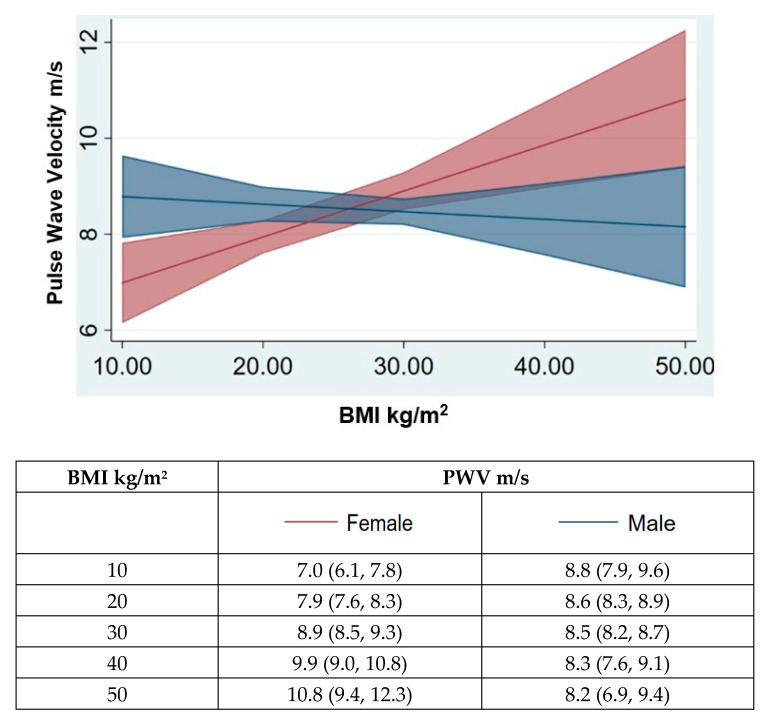
Age adjusted linear regression model with predictive margins to determine the association of BMI with arterial stiffening, measured by carotid-femoral pulse wave velocity (CF-PWV) in the study cohort. Predictive margin analysis indicated a PWV increase of 0.1m/s with every 1 kg/m^2^ increase in BMI with female subjects whereas this effect was not seen in male subjects.

The peripheral and central systolic BP levels were significantly elevated in the higher BMI ranges in females whereas no such differences were observed in the males with higher BMI. The brachial systolic BP was significantly higher in overweight (BP difference: Δ SBP 11 mmHg, *p* < 0.001) and obese (Δ SBP 9 mmHg, *p* < 0.001) females compared to their lean counterparts, while brachial BP remained the same across the different BMI groups in males (Table 1 and Table 2). Similarly, the central systolic BP was significantly (*p* < 0.05) higher in overweight (ΔSBP 10 mmHg) and obese (ΔSBP 8 mmHg) females compared to those in normal weight group, and no change was observed in the male subjects with higher BMI (Table 1 and Table 2). Predictive margin analysis revealed that in female subjects, every 1 kg/m^2^ increase in BMI was associated with an increase in PWV of 0.1m/s (Figure 1). No effect of BMI and PWV was observed in males.

### 3.3. Gender Based Associations of Central Anthropometric Measures with Arterial Damage

The influence of gender on the association of central anthropometric measures such as the waist–hip ratio (WHR) and waist–height ratio (WHtR) with arterial damage and BP elevation was assessed using correlation analysis. Pearson’s correlation showed that WHR and WHtR significantly correlated with CF-PWV in both males (*p* < 0.005) and females (*p* < 0.001) (Table 3). However, the central anthropometric measures significantly correlated with the central (WHR, r = 0.196, *p* < 0.005; WHtR, r = 0.271, *p* < 0.001) and peripheral (WHR, r = 0.202, *p* < 0.005; WHtR, r = 0.291, *p* < 0.001) systolic BP in the female subjects only and not in the males as shown in Table 3.

## 4. Discussion

In our study of the evaluation of sex related differences of obesity mediated vascular TOD in the Chinese cohort, body fat measures were strongly associated with aortic stiffness in females with significant positive association with the central and peripheral systolic BP compared to their male counterparts. The CF-PWV increased linearly with BMI - every 1 kg/m^2^ increase in BMI was associated with 0.1m/s increase in CF-PWV in female subjects whereas no such effect was observed in male subjects. These results demonstrate that excess body weight in females is associated with increased risk of vascular TOD, with the female gender being more susceptible to the development of hypertension in obesity, compared to the male counterparts. 

In addition, the uric acid levels, taken as an indicator of a pro-inflammatory milieu [27] were significantly associated with aortic stiffness in obese females (Table 3). The central anthropometric measures (WHR and WHtR) of abdominal obesity have been proposed to be better predictors of hemodynamic compromise and CV events [28,29,30]. Our finding demonstrated a significant correlation between WHR and WHtR with CF-PWV as well as central and brachial BP in females, unlike the male counterparts whose central anthropometric measures correlated only with aortic stiffness and not with pressure indices (Table 3).

Women are perceived to be protected from hypertension until menopausal ages [31]. This perceived dogma however, may be restricted to premenopausal lean women as hypertension is more heavily associated with increased BMI in obese young women [21,32]. The protection from the female sex hormones is possibly lost in obesity. Though carotid distensibility decreased with increasing BMI in both sexes at younger age [33], the PWV increased with higher BMI in middle-aged and older women but not in men [34]. Moreover, obesity associated insulin resistance and prediabetes ablates the cardio-protective effects of female sex hormones for both coronary heart disease and hypertension [35]. Data from studies are suggestive of desensitization effect of female sex hormones and the associated CV protective effects in obesity. In addition to the desensitisation of the oestrogen mediated vasoprotective effects in obesity, the male hormones, notably testosterone, are high in circulation of obese women, which further enhances the risk of CV morbidity [36]. Obesity induced insulin resistance is a stronger CV risk determinant in women compared to men [36]. The high fasting insulin levels associated with obesity mediated insulin resistance, in turn determine arterial stiffness and CV outcomes in women than men [36], while increasing insulin resistance was associated with greater increase in arterial stiffness in women and not in men [37]. 

### The Proposed Mechanism Predisposing the Female Gender to Obesity-Related Hypertension Compared to the Males

The renin angiotensin aldosterone system (RAAS) is an important regulator of vascular tone and BP, contributing to obesity related hypertension in a sex discrepant manner. The RAAS plays a significant role in the development of vascular remodelling and vascular TOD in obesity-associated hypertension in both men and women [38,39]. RAAS activation yields angiotensin II (Ang II), a potent vasoconstrictor and salt-retaining hormone and Ang II also stimulates adrenal aldosterone production through the canonical pathway, both of which are known to play a role in vascular remodelling [40]. The sex specificity of obesity-mediated RAAS activation is less explored. Healthy women subjects exhibited an unfavourable heart rate variability response (a measure of cardiac autonomic tone) and increased arterial stiffness to angiotensin II infusion whilst the male counterparts demonstrated an opposite effect [36] and the disruption of the non-canonical RAAS, Ang (1–7) pathway was identified to play a role in obesity-associated hypertension in obese female mice [41].

Increased aldosterone has been associated with hypertension and vascular dysfunction in obesity in males and females, outside of its role of renal sodium retention [42,43]. Aldosterone levels increase with parallel increase in adipose tissue and BMI more so in females than in males [44]. Moreover, adipocyte derived leptin stimulates aldosterone production, which promoted vascular dysfunction [45]. Female leptin-sensitive mice exhibited increased aldosterone levels, an effect that was absent in male counterparts, suggesting that females are specifically prone to leptin-induced aldosterone secretion in a sex-specific manner [45,46]. Studies in female animal models have demonstrated that vascular dysfunction and hypertension in obesity are mediated by leptin induced aldosterone secretion, whereas such an effect was not observed in, the male counterparts indicating that female gender is more prone to the development of leptin-mediated aldosterone induced vascular dysfunction and hypertension in females [47]. To note, obesity is often associated with hyperleptinemia [48]. This in line with the studies that have reported antagonism of aldosterone receptor (mineralocorticoid receptor) is more efficacious as a therapeutic cardiovascular regimen in women compared to men [49,50].

## 5. Conclusions

While much of the mechanistic studies are restricted to animal models, more research is required to delineate the causal pathways that predispose female gender to increased risk of obesity mediated vascular TOD in human subjects. From a clinical perspective, overweight and obesity in females is associated with increased CV risk resulting from arterial stiffening and higher BP, which may predispose to the development of obesity related hypertension compared to the overweight and obese male subjects. Though several possible mechanisms predisposing the female gender to increased obesity mediated arterial stiffening and hypertension have been proposed, clearly future studies are needed, looking into the specific causal mechanisms that could potentially guide gender specific clinical management of obesity related hypertension.

### Limitations

There are several limitations to be acknowledged in the current study. Firstly, this is a cross sectional study and there is the possibility of selection bias and we had no data on the menopausal status in female participants. Moreover, the relatively low number of certain subgroups, may lead to an underestimation of effects. The causal mechanisms such as RAAS activation, insulin resistance and others were not explored due to limited resources. However, this study included an otherwise healthy cohort to study vascular TOD using CF-PWV, a gold standard assessment of aortic stiffness that could provide us with robust data and which have advantages in terms of its general applicability in clinical settings. 

## Figures and Tables

**Table 1:** General characteristics of the study participants (mean ± SD).

	Study Participants	BMI < 24	BMI 24–28	BMI > 28	*p* Value between BMI Class
*n*	834	306	345	183	
Age (years)	54 (14)	55 (16)	55 (15)	51 (14)	
**Anthropometrics** BMI	25.5 (4)	22 (2)	26 (1)	31 (3)	
WC (cm)	91 (11)	83 (8)	92 (7)	102 (10)	*p* < 0.001
HC (cm)	98 (7)	93 (5)	98 (5)	105 (7)	*p* < 0.001
WHR	0.93 (0.08)	0.90 (0.08)	0.94 (0.07)	0.97 (0.08)	*p* < 0.001
WHtR	0.55 (0.06)	0.5 (0.05)	0.55 (0.04)	0.6 (0.06)	*p* < 0.001
**Arterial parameters**					
CF PWV (m/s)	8.5 (2.1)	8.2 (2.2)	8.6 (2.2)	8.6 (2.2)	*p* < 0.001
Brachial SP (mmHg)	134 (19)	131 (20)	135 (19)	135 (16)	*p* < 0.01
Brachial DP (mmHg)	77 (12)	73(12)	78 (12)	79 (11)	*p* < 0.001
Brachial PP (mmHg)	57 (14)	57 (15)	57 (13)	56 (13)	*p* < 0.001
Central SP (mmHg)	122 (19)	120 (20)	124 (20)	122 (16)	*p* < 0.05
Central DP (mmHg)	78 (12)	75 (12)	79 (13)	81 (11)	*p* < 0.001
Central PP (mmHg)	44 (13)	45 (14)	44 (13)	41 (12)	*p* = 0.0643
MAP (mmHg)	97 (14)	94 (15)	99 (15)	99 (13)	*p* < 0.001
CAIx (%)	139 (26)	28 (12)	26 (12)	21 (13)	*p* < 0.001
Systolic ejection duration	315 (26)	320 (25)	315 (24)	307 (27)	*p* < 0.001
End systolic pressure	110 (18)	107 (18)	111 (18)	110 (15)	*p* < 0.05
Biochemical Parameters					
Plasma glucose (mmol/L)	5.86 (1.9)	5.6 2 (1.7)	5.95 (1.8)	6.03 (2)	*p* < 0.05
**Liver profile**					
Alanine transaminase (ALT)	28.1 (27.8)	20 (11)	29 (19.7)	36.9 (23.4)	*p* < 0.001
Aspartate transaminase (AST)	25.6 (22.8)	22 (8.9)	26.8 (25.6)	29.5 (31)	*p* < 0.001
Gamma-glutamyl transferase	38.2 (36.9)	26.1 (24)	40 (33)	54.7 (52)	*p* < 0.001
**Renal profile** Blood urea nitrogen (BUN)	6.1 (12.5)	6.7 (20)	5.8 (3.8)	5.6 (1.5)	*p* = 0.458
Creatinine	79 (37.5)	78 (55.6)	79.33 (23)	79 (16.6)	*p* = 0.814
**Lipid Profile**					
Total Cholesterol (TC)	4.8 (1.13)	4.7 (1.1)	4.8 (1.2)	4.7 (1.2)	*p* = 0.644
Triglycerides (TGL)	2 (2.5)	1.5 (0.9)	2.23 (3.4)	2.4 (2.4)	*p* < 0.05
Low density lipoprotein (LDL) High density	3.4 (6.3)	3.1 (0.9)	3.8 (9.6)	3.3 (2.3)	*p* = 0.897
lipoprotein (HDL)	1.2 (0.5)	1.2 (0.4)	1.1 (0.7)	1.1 (0.4)	*p* < 0.005
**Inflammatory marker:** Uric acid (µmol/L)	359.4 (100.1)	321.9 (92.5)	374 (98)	394.39 (96.6)	*p* < 0.001

Table 1. Continuous clinical characteristics of the patient cohort. Data are given as mean ± SD. BMI, body mass index; WC, waist circumference; HC, hip circumference, WHR, waist–hip ratio; WHtR, waist–height ratio; CF PWV, carotid femoral pulse wave velocity; SP, systolic blood pressure; DP, diastolic blood pressure; PP, pulse pressure; MAP, mean arterial pressure; AIx; augmentation index.

**Table 2:** Arterial parameters (mean ± SD) grouped by age and sex (males and females).

Gender	Arterial Parameters	BMI Classes	Age in Years				*p* Value b/w BMI Classes	Post-hoc Analysis (Lean vs. Others BMI Classes)	*p* Value b/w Gender
			10–30	31–50	51–70	>71			
*n*			32	331	379	92			
Male	CF PWV	lean	6.6 (1.1)	7.4 (1.4)	9.0 (2.4)	10.5 (0.0)	*p* = 0.21	NS	*p* = 0.88
		overweight	7.6 (0.2)	7.7 (1.5)	9.0 (2.0)	10.4 (2.7)			
		obese	7.1 (2.2)	8.3 (1.5)	9.1 (2.0)	9.8 (1.7)			
			*p* = 0.82	*p* < 0.005	*p* = 0.90	*p* = 0.76			
Female		lean	5.5 (1.1)	7.2 (1.7)	8.2 (1.6)	10.2 (1.9)	*p* < 0.005		
		overweight	6.4 (0.5)	7.3 (0.8)	9.2 (1.9)	12.0 (3.5)		*p* < 0.005	
		obese	6.3 (0.2)	7.7 (1.4)	9.1 (2.3)	11.3 (1.9)		*p* < 0.005	
			*p* = 0.36	*p* = 0.67	*p* < 0.005	*p* = 0.25			
Male	Brachial SP	lean	133 (4.5)	131 (17.5)	136 (16.9)	135 (17.8)	*p* = 0.78	NS	*p* < 0.05
		overweight	143 (10.9)	131 (18)	137 (20)	135 (18.4)			
		obese	142 (20.2)	133 (14.1)	137 (17.0)	125 (15.0)			
			*p* = 0.70	*p* = 0.56	*p* = 0.93	*p* = 0.32			
Female		lean	116 (15.4)	123 (20.1)	129 (17.1)	148 (30.1)	*p* < 0.005		
		overweight	125 (18.7)	128 (19.5)	143 (18.1)	143 (26.1)		*p* < 0.005	
		obese	114 (12.7)	137 (16.8)	139 (18.7)	138 (7.9)		*p* < 0.05	
			*p* = 0.68	*p* = 0.16	*p* < 0.005	*p* = 0.74			
Male	Brachial DP	lean	77 (11.8)	77 (13.5)	79 (9.9)	70 (8.5)	*p* = 0.12	NS	*p* < 0.005
		overweight	81 (7.0)	79 (12.5)	78 (11.7)	73 (11.3)			
		obese	79 (14.8)	82 (11.7)	81 (10.0)	64 (7.2)			
			*p* = 0.94	*p* = 0.16	*p* = 0.50	*p* = 0.62			
Female		lean	70 (7.9)	72 (14.3)	71 (10.6)	75 (14.4)	*p* < 0.001		
		overweight	74 (20.9)	79 (13.0)	79 (13.0)	73 (10.5)		*p* < 0.005	
		obese	68 (13.4)	81 (9.4)	79 (9.1)	75 (6.0)		*p* < 0.005	
			*p* = 0.81	*p* = 0.07	*p* < 0.005	*p* = 0.89			
Male	Central SP	lean	118 (11.4)	118 (19.2)	126 (16.0)	124 (19.1)	*p* = 0.94	NS	*p* = 0.25
		overweight	125 (6.0)	119 (19.0)	126 (19.2)	123 (20.1)			
		obese	120 (16.9)	120 (13.5)	125 (16.5)	110 (13.0)			
			*p* = 0.82	*p* = 0.77	*p* = 0.94	*p* = 0.14			
Female		lean	104 (16.1)	114 (21.4)	119 (16.8)	138 (29.8)	*p* < 0.05		
		overweight	112 (21.0)	118 (20.0)	132 (17.4)	130 (25.0)		*p* < 0.005	
		obese	99 (17.7)	123 (18.6)	128 (19.6)	128 (8.4)		*p* < 0.05	
			*p* = 0.68	*p* = 0.37	*p* < 0.005	*p* = 0.62			
Male	Central DP	lean	78 (11.9)	78 (13.7)	80 (10.0)	71 (8.6)	*p* = 0.13	NS	*p* < 0.05
		overweight	82 (7.1)	80 (12.8)	79 (12.0)	74 (11.5)			
		obese	80 (15.0)	83 (11.7)	82 (10.2)	65 (7.8)			
			*p* = 0.95	*p* = 0.08	*p* = 0.44	*p* = 0.07			
Female		lean	71 (8.1)	74 (14.6)	72 (10.8)	76 (14.8)	*p* < 0.001		
		overweight	76 (21.5)	80 (13.1)	80 (13.1)	73 (11.0)		*p* < 0.005	
		obese	69 (12.7)	83 (9.5)	80 (9.4)	76 (5.8)		*p* < 0.005	
			*p* = 0.80	*p* = 0.06	*p* < 0.005	*p* = 0.86			

Data are given as mean ± SD. CF PWV, carotid femoral pulse wave velocity; SP, systolic blood pressure; DP, diastolic blood pressure; BMI, body mass index (kg/m^2^ ); lean, BMI < 24; overweight, BMI 24–28; obese, BMI > 28.

**Table 3:** Correlation analysis of WHR and WHeightR with pressure wave indices in males and females.

Central Anthropometric Measures	WHR		WHtR	
	M	F	M	F
CF PWV (m/s)	*p* = 0.004	*p* = 0.000	*p* = 0.003	*p* = 0.000
	r = 0.145	r = 0.386	r = 0.145	r = 0.412
Brachial SP (mmHg)	*p* = 0.372	*p* = 0.001	*p* = 0.063	*p* = 0.000
	r = −0.044	r = 0.202	r = 0.201	r = 0.291
Brachial PP (mmHg)	*p* = 0.651	*p* = 0.008	*p* = 0.55	*p* = 0.002
	r = −0.022	r = 0.166	r = −0.029	r = 0.198
Central SP (mmHg)	*p* = 0.146	*p* = 0.002	*p* = 0.54	*p* = 0.000
	r = −0.072	r = 0.196	r = 0.03	r = 0.271
Central PP (mmHg)	*p* = 0.239	*p* = 0.00	*p* = 0.109	*p* = 0.003
	r = −0.058	r = 0.174	r = −0.079	r = 0.185
Central MAP (mmHg)	*p* = 0.149	*p* = 0.023	*p* = 0.127	*p* = 0.000
	r = −0.071	r = 0.143	r = 0.075	r = 0.234

CF-PWV, carotid femoral pulse wave velocity; SP, systolic blood pressure; DP, diastolic blood pressure; PP, pulse pressure; MAP, mean arterial pressure; WHR, waist–hip ratio; WHtR, waist–height ratio.

## Data Availability

The data underlying this article cannot be shared publicly due to the privacy of individuals that participated in the study.
